# Contractions Induced in Human Pulmonary Arteries by a H_2_S Donor, GYY 4137, Are Inhibited by Low-Frequency (20 kHz) Ultrasound

**DOI:** 10.3390/biom14030257

**Published:** 2024-02-21

**Authors:** Agilė Tunaitytė, Silvijus Abramavičius, Augusta Volkevičiūtė, Mantas Venslauskas, Algimantas Bubulis, Vytis Bajoriūnas, Ulf Simonsen, Vytautas Ostaševičius, Vytautas Jūrėnas, Kasparas Briedis, Edgaras Stankevičius

**Affiliations:** 1 Preclinical Research Laboratory for Medicinal Products, Institute of Cardiology, Lithuanian University of Health Sciences, Sukileliu Ave. 13, 50166 Kaunas, Lithuania; silvijus.abramavicius@lsmu.lt (S.A.); augusta.volkeviciute@lsmu.lt (A.V.); vytis.bajoriunas@lsmu.lt (V.B.); edgaras.stankevicius@lsmu.lt (E.S.); 2 Institute of Physiology and Pharmacology, Lithuanian University of Health Sciences, A. Mickeviciaus st. 9, 44307 Kaunas, Lithuania; 3 Institute of Mechatronics, Kaunas University of Technology, Studentu st. 56, 51424 Kaunas, Lithuania; mantas.venslauskas@ktu.lt (M.V.); algimantas.bubulis@ktu.lt (A.B.); vytautas.ostasevicius@ktu.lt (V.O.); vytautas.jurenas@ktu.lt (V.J.); 4 Department of Biomedicine, Pulmonary and Cardiovascular Pharmacology, Aarhus University, Ole Worms Allé 4, 8000 Aarhus, Denmark; us@biomed.au.dk; 5 Institute of Cardiology, Lithuanian University of Health Sciences, Sukileliu Ave. 15, 50166 Kaunas, Lithuania; kasparas.briedis@lsmu.lt

**Keywords:** pulmonary hypertension, low-frequency (20 kHz) ultrasound, insonation, human pulmonary arteries

## Abstract

The present study aimed to investigate the effect of a H_2_S donor, GYY 4137, on human pulmonary arteries and whether low-frequency ultrasound (20 kHz, 4 W/cm^2^) inhibits GYY 4137 contractions. Functional studies were conducted on human and rat pulmonary arteries mounted on microvascular myographs. We placed an ultrasonic gadget in the tissue organ bath to insonate the arteries with low-frequency ultrasound. To measure the effect of the low-frequency ultrasound on the entrance of extracellular Ca^2+^, the preparations were placed in a Ca^2+^-free solution, and the thromboxane agonist, U46619, and extracellular calcium were added in the presence of insonation. In isolated human pulmonary arteries, GYY 4137 induced contractions, which were most pronounced in the arteries contracted with the thromboxane analogue, U46619. The transient GYY4137 contractions were reversed by low-frequency ultrasound, a blocker of KV_7_ channels, XE-991 (10 µM), and glibenclamide (1 μM), a blocker of ATP-sensitive channels. Low-frequency ultrasound also inhibited the contractions induced by the smooth muscle entrance of increasing extracellular calcium concentrations. The present findings show that GYY 4137 can cause a transient contraction of pulmonary arteries in human arteries. GYY 4137 alone does not cause significant vascular contraction in rat lung arteries, but it contracts rat lung arteries precontracted with U46619. The transient contractions induced by GYY 4137 can be inhibited by low-frequency ultrasound, probably by counteracting the influx of external Ca^2+^. The effect of low-frequency ultrasound counteracts contraction in pulmonary arteries; therefore, a possibility could be to develop a larger device allowing treatment of patients with pulmonary hypertension.

## 1. Introduction

Pulmonary hypertension (PH) is a condition that affects the lungs and causes an elevation in pulmonary artery pressure (with a mean pressure of at least 25 mmHg). This results in progressive vascular remodeling, which impairs the functional status and quality of life of individuals with PH. Unfortunately, PH is often associated with chronic lung disease (such as chronic obstructive pulmonary disease or idiopathic pulmonary fibrosis), and current treatment options are limited. Regrettably, no treatment is currently available that can completely halt the progression of PH [1].

Drug therapy for patients with PH has a different mechanism of action, e.g., endothelin receptor antagonists (ambrisentan), prostanoids, the prostaglandin I2 (IP) receptor agonist selexipag, phosphodiesterase type 5 inhibitors (tadalafil), and the soluble guanylate cyclase stimulator, riociguat [2]. Only interventional devices are available to treat PH: pulmonary artery denervation, right ventricular pacing, and mechanical circulatory support with durable ventricular assist devices [2,3].

H_2_S is a gasotransmitter synthesized in mammalian tissues akin to NO and carbon monoxide and induces vascular relaxation and contraction [4]. H_2_S-synthesizing enzymes, such as cystathionine gamma-lyase, cystathionine beta-synthase, and 3-mercaptopyruvate sulfur transferase, have been identified in lung tissue [5]. More importantly, the H_2_S levels and expression of CSE are lower in patients with PH [6], which leads to increased pulmonary blood flow and vascular structural remodeling in these patients [7]. Thus, H_2_S could ameliorate the excessive proliferation of the pulmonary arterial smooth muscle cells, reduce pulmonary vasoconstriction [8], and counteract endothelial inflammation [9]. GYY 4137 is a H_2_S donor and thus may have a potential therapeutic role in PH. However, H_2_S can also cause vascular contraction in rat aorta smooth muscle cells via Na^+^-K^+^-2Cl^−^ cotransport and L-type Ca^2+^ channels [10]. In addition, H_2_S can cause a two-phased contraction in rat pulmonary arteries (PAs), covering a first phase, which is a small short-term contraction followed by relaxation, and then a second phase with a larger and longer contraction via sulfide–quinone oxidoreductase-mediated sulfide metabolism, which, by giving electrons to ubiquinone, enhances electron production by complex III and thereby reactive oxygen species (ROS) production [11]. Thus, with our experiments, we aimed to test whether GYY 4137 can cause pulmonary vascular contraction in human lung vessels (an adverse effect on PH because such an effect should increase the pulmonary artery pressure) and see whether this could be inhibited with low-frequency ultrasound (LUS). If LUS can counteract the GYY 4137-induced vascular contraction, the drug–device combination of GYY 4137 with LUS can diminish the adverse effect of vascular contraction and promote the beneficial properties of H_2_S, for example, facilitating the inhibition of endothelial inflammation [9].

Ultrasound has been used in medicine for diagnostics and therapy alike. While the thermal effects of ultrasound have been known for decades, more recently, the non-thermal effects have attracted more attention from researchers [12]. LUS has therapeutic effects mainly attributed to non-thermal factors that result in the formation of microbubbles and microjets through cavitation, mechanical stimulation, and acoustic streaming. These factors have diverse biological consequences, including the regulation of cell proliferation and differentiation through the activation of protein expression such as Runt-related transcription factor 2 (Runx2) and the phosphorylation of extracellular signal-regulated kinase 1/2 (ERK1/2) and p38 mitogen-activated protein kinase (p38 MAPK). Additionally, these effects can lead to the opening of membrane channels, such as the activation of BK(Ca) channels [13,14]. LUS has been shown to alter iNOS expression [15,16]. Thus, there is potential evidence that LUS can affect gas transmitters like NO and H_2_S [17].

We have previously found that LUS inhibits dopamine-induced vascular contraction in rat mesenteric arteries (precontracted with KPSS) and promotes dopaminergic vascular contraction in human pulmonary arteries immersed in KPSS. In contrast, other authors have shown various therapeutic properties of ultrasound, including facilitation of vascular relaxation [17,18]. These findings lead us to believe that LUS can also change drug action in human pulmonary vessels.

In previous preliminary studies, we observed that hydrogen sulfide salts induced contraction followed by relaxation in pulmonary arteries [19]. GYY 4137 is a stable hydrogen sulfide donor causing relaxation of rat mesenteric arteries [17]. The present study aimed to investigate the effect of GYY 4137 in human pulmonary arteries and whether LUS inhibited GYY 4137 contractions. We gave a preliminary report, published as an abstract, on some of the data presented in this manuscript at the RSU International Research Conference on Medical and Health Care Sciences, Riga, 2021.

## 2. Materials and Methods

### 2.1. Chemicals and Materials

The drugs used: glibenclamide, GYY 4137, U46619, tadalafil, ambrisentan, and XE-991 were from Sigma-Aldrich (St. Louis, MO, USA). The physiologic salt solution (PSS) comprised NaCl 119 mM, NaHCO_3_ 25 mM, glucose 5.5 mM, CaCl_2_ 1.6 mM, KH_2_O_4_ 1.18 mM, MgSO_4_ 1.17 mM, and EDTA 0.027 mM (all from Sigma-Aldrich, St. Louis, MO, USA). The Ca^2+^-free PSS solution was identical to the PSS solution except for excluding CaCl_2_. The 119 mM K^+^ solution is a physiological saline solution with a high potassium concentration (KPSS) that had the same composition as the PSS but with the NaCl replaced by KCl on an equimolar basis to reach a final K^+^ concentration of 119 mM.

### 2.2. LUS

The ultrasonic device was immersed in the tissue organ bath so that the vessel would be insonated externally, as the current hypothesis is that LUS can produce identifiable biological effects in vessel tissues without requiring intravascular access. The ultrasound generator VT-400 has a supply voltage of 200–240 V and output power of up to 400 W. It operates in an output frequency between 15 and 60 kHz (a 20 kHz frequency was used during the experimental procedures). The acoustic power density was 4 W/cm^2^ at 20.33 kHz. This corresponds to a mechanical index (MI) of 2.43, above what the safe threshold would be if administered intraluminally [20].

### 2.3. Functional Studies in Pulmonary Arteries

Human pulmonary arteries were dissected from the vascular bed and mounted on 40 µm steel wires in the myographs (Danish Myotechnology, Aarhus, Denmark) for isometric tension recording, as previously described [21]. The vessels were equilibrated in oxygenated (5% CO_2_, 20% O_2_, 75% N_2_) PSS at 37 °C for 30 min and, by stretching, normalized to a lumen diameter (d100) equivalent to 100 mm Hg (23 mm Hg in human pulmonary arteries), after which the tension was set to 90% × d100 [21]. After normalization, the arterial segments were stimulated with KPSS, washed in PSS, and stimulated with 10 µM NA. The mechanical responses of the vessel segments were measured as the active wall tension (ΔT), which is the change in force (ΔF) divided by twice the segment length [18]. An identical protocol was used for rat pulmonary arteries.

### 2.4. Experimental Procedures

We incubated pulmonary arteries with GYY 4137 to test whether it could cause vascular contraction and attempted to modulate this effect with LUS. We tested Ca^2+^ signaling in human pulmonary vessels by contracting the vessels with U46619 (a thromboxane A2 mimetic that binds to specific G-protein-coupled receptors (TP receptors) [22] in Ca^2+^ free PSS, constructed a CaCl_2_ contraction dose–response curve, and modulated this effect with LUS [18]. We tested the phosphodiesterase type 5 (PDE5) inhibition effect on H_2_S signaling by incubating vessels in tadalafil (1 µmol) and that of endothelin-1 by incubating in ambrisentan (1 µmol) [22,23] and modulate it with LUS. We also tested the involvement of potassium channels in the GYY 4137-induced pulmonary vascular contraction: we used glibenclamide to inhibit KATP channels [24] and the K_V_7.2/7.3 blocker XE-991 [25].

### 2.5. Data and Statistical Analysis

The data were expressed as the mean ± standard error of the mean (S.E.M.) or standard deviation (SD) with a significance level of *p* < 0.05; n represents the number of individuals. A two-way analysis of variance was used to compare the means of functional studies observations. Graphs were created, and statistical analyses were performed using the SAS University Edition 2014–2021 (SAS OnDemand for Academics|SAS, 2014), R-4.3.2 and Microsoft Excel (Microsoft 365, Kaunas, Lithuania).

### 2.6. Group Size

Each experiment was performed at least five times on vessels harvested from lungs obtained from patients undergoing pneumonectomy due to lung cancer unless stated otherwise.

## 3. Results

### 3.1. Effect of LUS on GYY 4137-Induced Vascular Contractions

**Figure 1:**
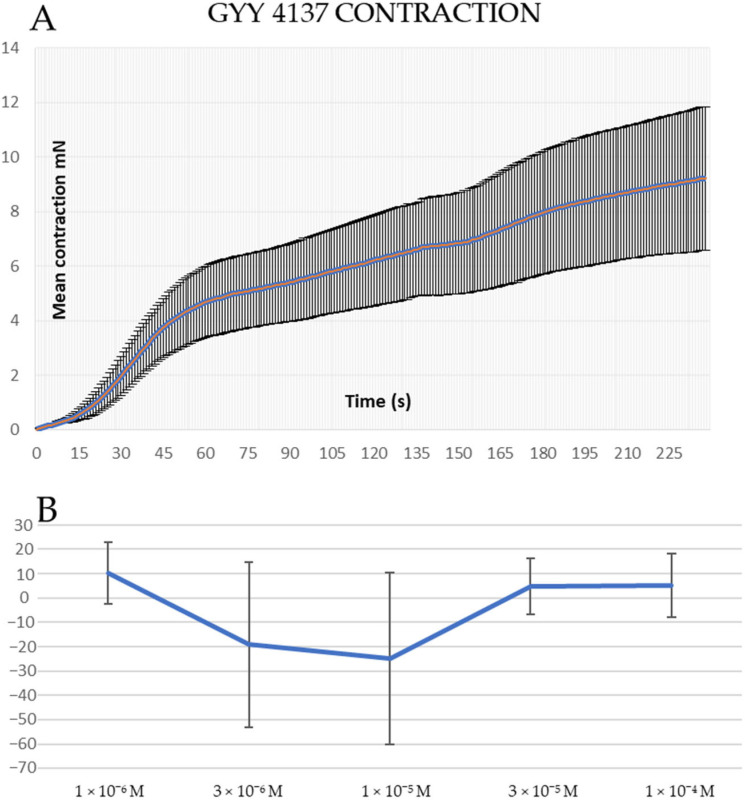
(**A**)—Average contraction induced by 10^−4^ M GYY 4137 in human pulmonary arteries. The data are mean ± standard error of the mean (S.E.M.), *n* = 3. (**B**)—Concentration–response curve for GYY 4137 in rat lung arteries. Data points are means ± S.E.M. of 5 preparations.

GYY 4137 induced contractions in human pulmonary arteries when added at baseline (Figure 1A). We repeated a similar experiment in rat lung arteries and found that adding increasing concentrations of GYY 4137 did not elicit vascular contraction at baseline (Figure 1B).

**Figure 2:**
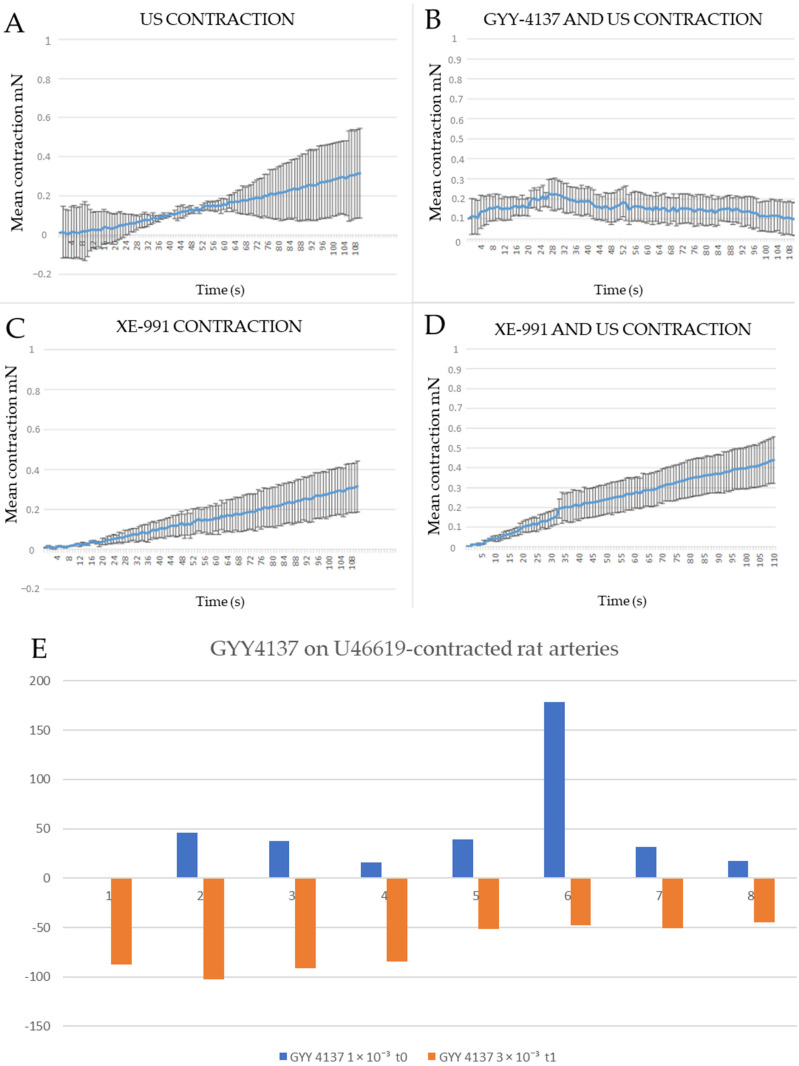
Baseline contractions (**A**–**D**), *n* = 3 (for each graph): (**A**). Ultrasound (US) does not significantly change the function of normalized pulmonary arteries. (**B**). After GYY 4137 precontraction, additional insonation causes no discernable effect. (**C**). XE-991 does not cause vascular contraction in resting pulmonary arteries. (**D**). XE-991 and US do not cause vascular contraction in normalized pulmonary arteries. (**E**). Adding GYY 4137 to rat lung U46619-contracted rat arteries (*n* = 7) elicits a biphasic response. Mean 45.8 (SD 55.5) at t_0_ (which lasts for about 1 min after adding GYY 4137) and a relaxation −70.0 % (SD 23.4) from t_1_ to later time points.

In contrast, ultrasound, XE-991 incubation, and GYY4137 with insonation produced negligible effects on the vascular tone (up to 1 mN) (Figure 2).

**Figure 3:**
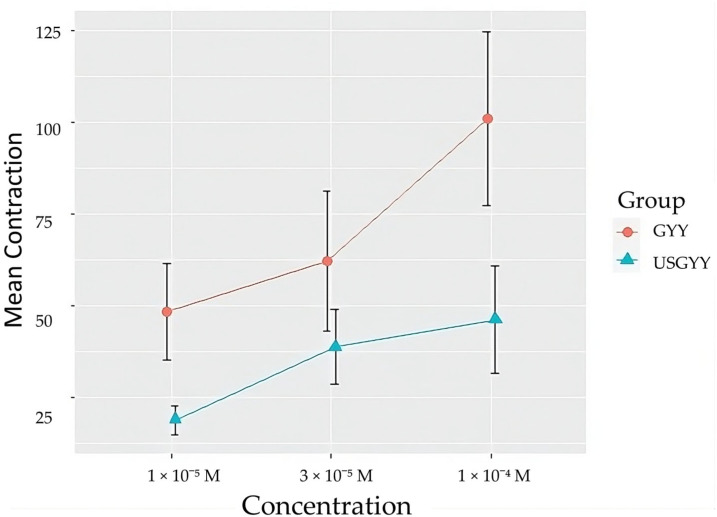
Concentration–response curve for GYY4137 in the absence (control) or in the presence of insonation (GYY + insonation). Two-way (class factors: groups and concentration) analysis of variance (ANOVA): F (8) = [3.22, 10.86], *p* = [0.1106, 0.0052], n = 5 per group. The graphs represent the vascular response to increasing concentrations of GYY4137. Data are represented as mean ± S.E.M.

GYY 4137 also induces small vascular relaxations in U46619-contracted arteries after a brief vascular contraction in rat pulmonary arteries (Figure 1B and Figure 2B). Similar effects can be seen in human pulmonary arteries, and this effect can be partially reversible with insonation in human pulmonary arteries (see Figure 3). This effect can also, in part, be caused by the spontaneous contraction of human pulmonary arteries.

### 3.2. Potassium Channel Involvement in the GYY 4137-Mediated Pulmonary Vascular Contraction

**Figure 4:**
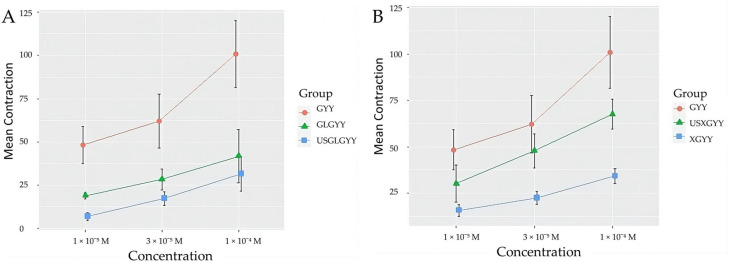
Average contractions induced by GYY 4137 in the absence and the presence of the potassium channel blockers, glibenclamide and XE991. (**A**)—Average GYY 4137 contraction in the absence (GYY) and the presence of a blocker of ATP-sensitive potassium channels, glibenclamide (GLGYY) and glibenclamide plus low-frequency ultrasound (USGLGYY) compared with each other with two-way (class factors: groups and concentration) analysis of variance (ANOVA): F (12) = [5.25, 7.77], *p* = [0.0231, 0.0069], *n* = 5 per group. (**B**)—GYY 4137 induced vascular contraction in the absence (GYY) and the presence of a blocker of K_V_7 channels, XE-991 incubation (XGYY) and XE991 plus low-frequency ultrasound (USXGYY), compared with each other with two-way (class factors: groups and concentration) analysis of variance (ANOVA): F (12) = [3.73, 21.93], *p* = [0.0550, <0.0001], *n* = 5 per group. The graphs represent the vascular response to increasing concentrations of GYY4137. Data are represented as mean ± S.E.M.

Our results indicate that the GYY 4137-induced contraction of pulmonary vessels was blocked with XE-991, a KCNQ (K_V_7.2/7.3) inhibitor, and glibenclamide, an inhibitor of vascular ATP-sensitive potassium (K_ATP_) channels. LUS changed the activity of the K_V_7.2/7.3 by counteracting the effects of the XE-991 and potentiating the inhibition with glibenclamide (Figure 4).

Our findings show that K_V_7 and K_ATP_-mediated vascular effects elicited with a H_2_S donor GYY 4137 can be modulated with LUS, and LUS can potentially affect these channels.

### 3.3. Ca^2+^ Signaling in Human Pulmonary Vessels

**Figure 5:**
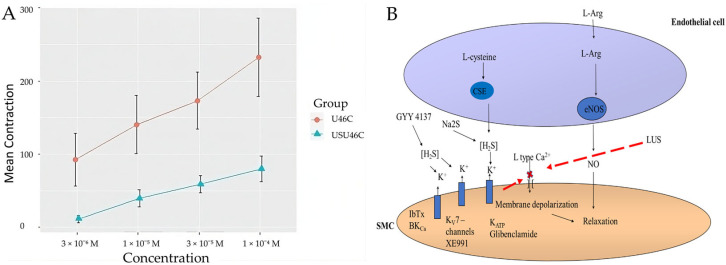
(**A**)—U46619 and CaCl_2_ induced vascular contraction in the absence (U46C) or in the presence of insonation (USU46C). Two-way (class factors: groups and concentration) analysis of variance (ANOVA): F (8) = [5.66, 21.64], *p* = [0.0489, 0.0006], n = 5 per group. The graphs represent the average vascular responses to increasing concentrations of extracellular CaCl_2_. Data are represented as mean and S.E.M. (**B**)—Proposed mechanism of GYY 4137 vascular contraction and insonation.

A thromboxane A2 (TP) receptor agonist, U46619, was used to contract human pulmonary arteries in the PSS without Ca^2+^. Subsequently, CaCl_2_ solution was added in increasing concentrations from 3 × 10^−6^ M to 1 × 10^−4^ M. During insonation, the insonated vessels showed a smaller contraction (Figure 5).

### 3.4. Phosphodiesterase Type 5 (PDE5) Inhibition and H_2_S Signaling

**Figure A1:**
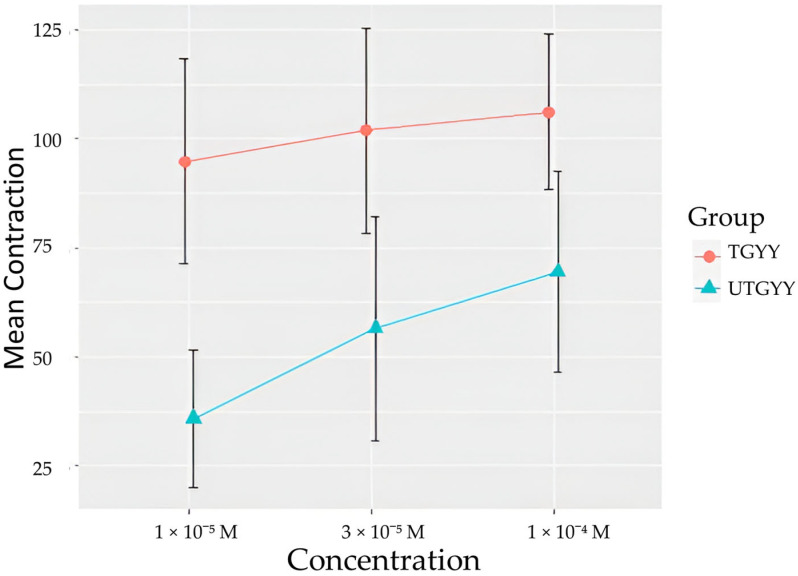
GYY 4137 induced vascular contraction interacting with increasing concentrations of tadalafil insonation (UTGYY) vs. control (TGYY). Two-way (class factors: groups and concentration) analysis of variance (ANOVA): F (10) = [2.96, 5.37], *p* = [0.1161, 0.0260], *n* = 6 per group. The graphs represent the vascular response to increasing concentrations of tadalafil in vessels contracted with GYY 4137 (1 × 10^−4^ M). These concentrations are used for exploratory purposes and are above therapeutic concentrations. Data are represented as mean and S.E.M.

A concentration–response curve for the phosphodiesterase type 5 (PDE5) inhibitor was constructed in human pulmonary vessels contracted with GYY 4137. The inhibition of PDE5 did not reverse the contraction, and insonation did not appear to potentiate the effect of tadalafil on the human pulmonary artery. Compared to the non-insonated vessels, the insonated vessel contracted at a lower rate (Figure A1).

### 3.5. Endothelin Receptors and H_2_S Signaling

**Figure A2:**
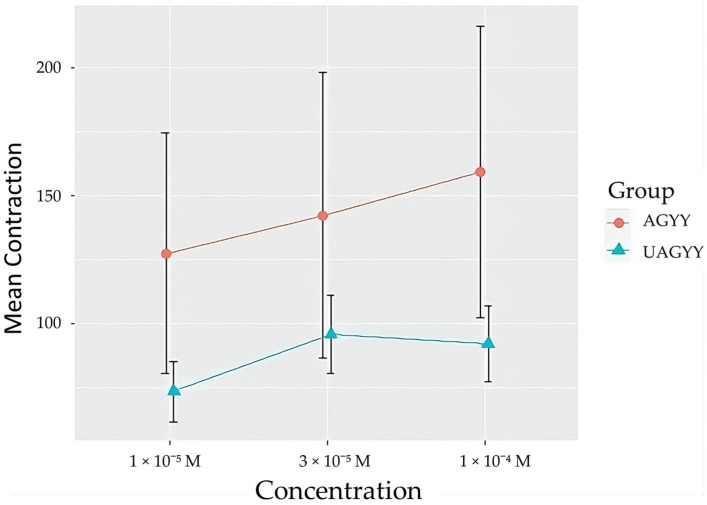
GYY 4137 induced vascular contraction interacting with increasing concentrations of ambrisentan insonation (UAGYY) vs. control (AGYY). Two-way (class factors: groups and concentration) analysis of variance (ANOVA): F (10) = [1.25, 11.39], *p* = [0.2904, 0.0026], *n* = 6 per group. The graphs represent the vascular response to increasing concentrations of ambrisentan in vessels contracted with GYY 4137 (1 × 10^−4^ M). Data are represented as mean and S.E.M.

In GYY 4137-contracted human pulmonary vessels, a selective endothelin receptor agonist ambrisentan was used, and concentration–response curves were constructed. With ambrisentan, the contraction was not reversed. Compared to the control vessels, insonated vessels with ambrisentan produced more significant contractions (Figure A2).

## 4. Discussion

H_2_S stimulates the K_ATP_ channels [26] and voltage-sensitive potassium channels (K_V_ channels) [27] but inhibits the inwardly rectifying potassium (Kir) channels (Kir2 and Kir3) and calcium-independent transient outward potassium current (Ito) channels [28]. The inwardly rectifying K^+^ channels significantly contribute to flow-induced vasodilatation in resistance arteries [29] and are functionally related to the K_V_7 channels [30]. GYY 4137 is a H_2_S donor; so, inhibiting the inwardly rectifying potassium channels can explain the observed vascular contraction. We also found that GYY 4137-induced pulmonary vasoconstriction can be reduced with LUS and that this effect can be explained by the reduced influx of the extracellular Ca^2+^ because the LUS reduces the vascular contraction to CaCl_2_ in the vessels incubated with U46619 in the PSS without Ca^2+^.

Salts of hydrogen sulfide, e.g., Na_2_S and NaHS, were found to counteract the development of pulmonary hypertension in chronic hypoxic rats [31] and monocrotaline-exposed rats [32]. In pulmonary arteries, Na_2_S and NaHS induced contraction followed by relaxation [19]. Therefore, we cannot exclude lower concentrations of GYY 4137, which will induce relaxation and may positively affect pulmonary hypertension.

Our study reveals that both tadalafil, which inhibits Phosphodiesterase type 5 (PDE5), and ambrisentan, which inhibits selective endothelin receptors, do not induce vasorelaxation in contracted human lung vessels treated with GYY 4137. Furthermore, our findings indicate that the insonated arteries contract to a lesser extent than control vessels. Some authors report that NO production attenuates H_2_S-mediated vascular responses, including vasorelaxation and angiogenesis, while H_2_S can inhibit NO-mediated vascular functions [33]. We show similar results, as it seems that the presence of H_2_S inhibits tadalafil’s nitric oxide-mediated vasodilatation in GYY 4137-contracted arteries.

We found that XE-991 nullified the GYY 4137-induced transient contraction of pulmonary vessels and that this effect can be prevented with LUS. XE-991 modulates the K_V_7 channels [25] and ERG (K_V_11.1–11.3), rectifying the ion channels [34]. On the one hand, activation of the K_V_ channels, which is part of the negative feedback regulation of the vascular tone, usually reduces vascular contraction; however, the K_V_ channel closure plays a part in the mechanism by which vasoconstrictor substances such as phenylephrine, serotonin, and angiotensin II act [35]. On the other hand, the K_V_7 channel activation can exert bimodal effects on vascular potassium currents; both effects are blocked with the K_V_7 blocker XE-991 [36]. It seems that active K_V_7 channels are needed to produce GYY 4137 vascular contraction in human pulmonary arteries. The LUS reverses the effect of the XE-991 on the GYY 4137-elicited contraction.

We have shown that glibenclamide inhibits GYY 4137-induced transient contraction of pulmonary vessels and that this effect is enhanced with LUS. It also has been shown that glibenclamide relaxes vascular smooth muscle constriction and that this action is not mediated by the cGMP or ATP-sensitive potassium channels [37]. Glibenclamide has dual effects on relaxation. It promotes endothelium-dependent relaxation by releasing nitric oxide and endothelium-independent relaxation by inhibiting Ca^2+^ influx through Ca^2+^ channels and the protein kinase C pathway [38]. This counteracts the vascular relaxation induced by GYY 4137.

## 5. Conclusions

The present findings show that GYY 4137 induces contraction in human pulmonary arteries, while in rat pulmonary arteries, transient contractions are only observed in preparations contracted with U46619. Contractions induced by GYY 4137 are inhibited by low-frequency ultrasound, probably by counteracting the influx of external Ca^2+^. The effect of low-frequency ultrasound counteracts the contraction in pulmonary arteries; therefore, a possibility could be to develop a larger device allowing treatment of patients with pulmonary hypertension.

## Data Availability

The data presented in this study are available in this article.

## References

[1] Elia D., Caminati A., Zompatori M., Cassandro R., Lonati C., Luisi F., Pelosi G., Provencher S., Harari S. (2019). Pulmonary hypertension and chronic lung disease: Where are we headed?. Eur. Respir. Rev..

[2] Ghofrani H.A., Grünig E., Jansa P., Langleben D., Rosenkranz S., Preston I.R., Rahaghi F., Sood N., Busse D., Meier C. (2020). Efficacy and safety of riociguat in combination therapy for patients with pulmonary arterial hypertension (PATENT studies). Pulm. Circ..

[3] Khan S.S., Rich J.D. (2015). Novel technologies and devices for monitoring and treating pulmonary arterial hypertension. Can. J. Cardiol..

[4] Skovgaard N., Olson K.R. (2012). Hydrogen sulfide mediates hypoxic vasoconstriction through a production of mitochondrial ROS in trout gills. Am. J. Physiol. Regul. Integr. Comp. Physiol..

[5] Olson K.R., Whitfield N.L., Bearden S.E., St Leger J., Nilson E., Gao Y., Madden J.A. (2010). Hypoxic pulmonary vasodilation: A paradigm shift with a hydrogen sulfide mechanism. Am. J. Physiol. Regul. Integr. Comp. Physiol..

[6] Meng G., Ma Y., Xie L., Ferro A., Ji Y. (2015). Emerging role of hydrogen sulfide in hypertension and related cardiovascular diseases. Br. J. Pharmacol..

[7] Xiaohui L., Junbao D., Lin S., Jian L., Xiuying T., Jianguang Q., Bing W., Hongfang J., Chaoshu T. (2005). Down-regulation of endogenous hydrogen sulfide pathway in pulmonary hypertension and pulmonary vascular structural remodeling induced by high pulmonary blood flow in rats. Circ. J..

[8] Yao Z., Wang C. (2019). A Novel Mechanism of Sildenafil Improving the Excessive Proliferation and H_2_S Production in Pulmonary Arterial Smooth Muscle Cells. J. Cardiovasc. Pharmacol..

[9] Feng S., Chen S., Yu W., Zhang D., Zhang C., Tang C., Du J., Jin H. (2017). H_2_S inhibits pulmonary arterial endothelial cell inflammation in rats with monocrotaline-induced pulmonary hypertension. Lab. Investig..

[10] Orlov S.N., Gusakova S.V., Smaglii L.V., Koltsova S.V., Sidorenko S.V. (2017). Vasoconstriction triggered by hydrogen sulfide: Evidence for Na^+^,K^+^,2Cl-cotransport and L-type Ca^2+^ channel-mediated pathway. Biochem. Biophys Rep..

[11] Prieto-Lloret J., Snetkov V.A., Shaifta Y., Docio I., Connolly M.J., Mackay C.E., Knock G.A., Ward J.P.T., Aaronson P.I. (2018). Role of reactive oxygen species and sulfide-quinone oxoreductase in hydrogen sulfide-induced contraction of rat pulmonary arteries. Am. J. Physiol.-Lung Cell Mol. Physiol..

[12] Jiang X., Savchenko O., Li Y., Qi S., Yang T., Zhang W., Chen J. (2019). A Review of Low-Intensity Pulsed Ultrasound for Therapeutic Applications. IEEE Trans. Biomed. Eng..

[13] Juffermans L.J.M., Kamp O., Dijkmans P.A., Visser C.A., Musters R.J.P. (2008). Low-intensity ultrasound-exposed microbubbles provoke local hyperpolarization of the cell membrane via activation of BK(Ca) channels. Ultrasound Med. Biol..

[14] Lin G., Reed-Maldonado A.B., Lin M., Xin Z., Lue T.F. (2016). Effects and Mechanisms of Low-Intensity Pulsed Ultrasound for Chronic Prostatitis and Chronic Pelvic Pain Syndrome. Int. J. Mol. Sci..

[15] Ichijo S., Shindo T., Eguchi K., Monma Y., Nakata T., Morisue Y., Kanai H., Osumi N., Yasuda S., Shimokawa H. (2021). Low-intensity pulsed ultrasound therapy promotes recovery from stroke by enhancing angio-neurogenesis in mice in vivo. Sci. Rep..

[16] Yuan L.J., Niu C.C., Lin S.S., Yang C.Y., Chan Y.S., Chen W.J., Ueng S.W. (2014). Effects of low-intensity pulsed ultrasound and hyperbaric oxygen on human osteoarthritic chondrocytes. J. Orthop. Surg. Res..

[17] Abramavicius S., Petersen A.G., Renaltan N.S., Prat-Duran J., Torregrossa R., Stankevicius E., Whiteman M., Simonsen U. (2021). GYY4137 and Sodium Hydrogen Sulfide Relaxations Are Inhibited by L-Cysteine and KV7 Channel Blockers in Rat Small Mesenteric Arteries. Front. Pharmacol..

[18] Abramavičius S., Volkevičiūtė A., Tunaitytė A., Venslauskas M., Bubulis A., Bajoriūnas V., Stankevičius E. (2020). Low-Frequency (20 kHz) Ultrasonic Modulation of Drug Action. Ultrasound Med. Biol..

[19] Skovgaard N., Gouliaev A., Aalling M., Simonsen U. (2011). The Role of Endogenous H_2_S in Cardiovascular Physiology. Curr. Pharm. Biotechnol..

[20] Abramavičius S., Volkevičiūtė A., Tunaitytė A., Venslauskas M., Bubulis A., Bajoriūnas V., Kowalski I.M., Stankevičius E. Ultrasonic modulation of drug action. Proceedings of the 3rd Meeting of the Baltic Physiological Societies.

[21] Mulvany M.J., Halpern W. (1976). Mechanical properties of vascular smooth muscle cells in situ. Nature.

[22] McKenzie C., MacDonald A., Shaw A.M. (2009). Mechanisms of U46619-induced contraction of rat pulmonary arteries in the presence and absence of the endothelium. Br. J. Pharmacol..

[23] Humbert M., Guignabert C., Bonnet S., Dorfmüller P., Klinger J.R., Nicolls M.R., Olschewski A.J., Pullamsetti S.S., Schermuly R.T., Stenmark K.R. (2019). Pathology and pathobiology of pulmonary hypertension: State of the art and research perspectives. Eur. Respir. J..

[24] Ripoll C., Jon Lederer W., Nichols C.G. (1993). On the Mechanism of Inhibition of KATP Channels by Glibenclamide in Rat Ventricular Myocytes. J. Cardiovasc. Electrophysiol..

[25] Zhang L., Wang Y., Li Y., Li L., Xu S., Feng X., Liu S. (2018). Hydrogen sulfide (H_2_S)-releasing compounds: Therapeutic potential in cardiovascular diseases. Front. Pharmacol..

[26] Ha J., Xu Y., Kawano T., Hendon T., Baki L., Garai S., Papapetropoulos A., Thakur G.A., Plant L.D., Logothetis D.E. (2018). Hydrogen sulfide inhibits Kir2 and Kir3 channels by decreasing sensitivity to the phospholipid phosphatidylinositol 4,5-bisphosphate (PIP2). J. Biol. Chem..

[27] Lv B., Chen S., Tang C., Jin H., Du J., Huang Y. (2021). Hydrogen sulfide and vascular regulation—An update. J. Adv. Res..

[28] Ma S.F., Luo Y., Ding Y.J., Chen Y., Pu S.X., Wu H.J., Wang Z.F., Tao B.B., Wang W.W., Zhu Y.C. (2015). Hydrogen Sulfide Targets the Cys320/Cys529 Motif in Kv4.2 to Inhibit the Ito Potassium Channels in Cardiomyocytes and Regularizes Fatal Arrhythmia in Myocardial Infarction. Antioxid. Redox Signal..

[29] Ahn S.J., Fancher I.S., Bian J.T., Zhang C.X., Schwab S., Gaffin R., Phillips S.A., Levitan I. (2017). Inwardly rectifying K+ channels are major contributors to flow-induced vasodilatation in resistance arteries. J. Physiol..

[30] Li R., Andersen I., Aleke J., Golubinskaya V., Gustafsson H., Nilsson H. (2013). Reduced anti-contractile effect of perivascular adipose tissue on mesenteric small arteries from spontaneously hypertensive rats: Role of Kv7 channels. Eur. J. Pharmacol..

[31] Wang R. (2012). Physiological implications of hydrogen sulfide: A whiff exploration that blossomed. Physiol. Rev..

[32] Turhan K., Alan E., Yetik-Anacak G., Sevin G. (2022). H_2_S releasing sodium sulfide protects against pulmonary hypertension by improving vascular responses in monocrotaline-induced pulmonary hypertension. Eur. J. Pharmacol..

[33] Kolluru G.K., Shen X., Kevil C.G. (2013). A tale of two gases: NO and H_2_S, foes or friends for life?. Redox Biol..

[34] Elmedyb P., Calloe K., Schmitt N., Hansen R.S., Grunnet M., Olesen S.P. (2007). Modulation of ERG channels by XE-991. Basic. Clin. Pharmacol. Toxicol..

[35] Tykocki N.R., Boerman E.M., Jackson W.F. (2017). Smooth Muscle Ion Channels and Regulation of Vascular Tone in Resistance Arteries and Arterioles. Compr. Physiol..

[36] Yeung S.Y.M., Schwake M., Pucovský V., Greenwood I.A. (2008). Bimodal effects of the Kv7 channel activator retigabine on vascular K+ currents. Br. J. Pharmacol..

[37] Zhang H., Stockbridge N., Weir B., Krueger C., Cook D. (1991). Glibenclamide relaxes vascular smooth muscle constriction produced by prostaglandin F2α. Eur. J. Pharmacol..

[38] Chan W.K., Yao X., Ko W.H., Huang Y. (2000). Nitric oxide mediated endothelium-dependent relaxation induced by glibenclamide in rat isolated aorta. Cardiovasc. Res..

